# Interaction of detergents with biological membranes: Comparison of fluorescence assays with filtration protocols and implications for the rates of detergent association, dissociation and flip-flop

**DOI:** 10.1371/journal.pone.0222932

**Published:** 2019-10-16

**Authors:** Philippe Champeil, Béatrice de Foresta, Martin Picard, Carole Gauron, Dominique Georgin, Marc le Maire, Jesper V. Møller, Guillaume Lenoir, Cédric Montigny

**Affiliations:** 1 Institute for Integrative Biology of the Cell (I2BC), CEA, CNRS, Université Paris-Sud, Université Paris-Saclay, Gif-sur-Yvette, France; 2 CEA, DSV, Institut des Sciences du Vivant Frédéric Joliot, Service de Chimie Biorganique et de Marquage, Gif-sur-Yvette, France; 3 Center for Membrane Pumps in Cells and Disease, PUMPKIN, Danish National Research Foundation, Aarhus University, Aarhus, Denmark; 4 Department of Biomedicine, Aarhus University, Aarhus, Denmark; University of Michigan, UNITED STATES

## Abstract

The present study mainly consists of a re-evaluation of the rate at which C_12_E_8_, a typical non-ionic detergent used for membrane studies, is able to dissociate from biological membranes, with sarcoplasmic reticulum membrane vesicles being used as an example. Utilizing a brominated derivative of C_12_E_8_ and now stopped-flow fluorescence instead of rapid filtration, we found that the rate of dissociation of this detergent from these membranes, merely perturbed with non-solubilizing concentrations of detergent, was significantly faster (t_1/2_ < 10 ms) than what had previously been determined (t_1/2_ ~300–400 ms) from experiments based on a rapid filtration protocol using ^14^C-labeled C_12_E_8_ and glass fiber filters *(Binding of a non-ionic detergent to membranes*: *flip-flop rate and location on the bilayer*, *by Marc le Maire*, *Jesper Møller and Philippe Champeil*, *Biochemistry (1987) Vol 26*, *pages 4803–4810)*. We here pinpoint a methodological problem of the earlier rapid filtration experiments, and we suggest that the true overall dissociation rate of C_12_E_8_ is indeed much faster than previously thought. We also exemplify the case of brominated dodecyl-maltoside, whose kinetics for overall binding to and dissociation from membranes comprise both a rapid and a sower phase, the latter being presumably due to flip-flop between the two leaflets of the membrane. Consequently, equilibrium is reached only after a few seconds for DDM. This work thereby emphasizes the interest of using the fluorescence quenching associated with brominated detergents for studying the kinetics of detergent/membrane interactions, namely association, dissociation and flip-flop rates.

## Introduction

Since the early seventies, natural membranes were used to study interactions of membrane proteins and lipids with detergents [1–3]. Sarco-endoplasmic reticulum vesicles (SR) prepared from fast-twitch muscle contain the Sarco-Endoplasmic Reticulum Ca^2+^-ATPase isoform 1a (SERCA1a) at very high density and purity, making this natural sample of particular interest for studying the effect of detergent on a membrane protein [4–6]. In 1984, Ueno, Tanford and Reynolds reported that when reconstituting membranes from detergent-solubilized SR, and using polystyrene hydrophobic beads for trapping of the non-ionic detergent octaethylene-glycol-dodecylether (C_12_E_8_), it was virtually impossible to get rid of almost half of the initially bound C_12_E_8_. The authors then suggested that this particular detergent experienced an extremely slow flip-flop (over a period of days) from one side of the membrane to the other [7]. At variance, in subsequent experiments in our laboratory, performed using a rapid filtration unit for measuring the kinetics of detergent removal from biological membrane vesicles or pure lipid vesicles, previously deposited on glass fiber filters, C_12_E_8_ removal from such vesicles was found to reach a state of complete removal with an apparent half-time of 300–400 ms, and similar results were also obtained with multi-layered lipid vesicles (Fig 5 of ref. [8]). It was therefore concluded that the results of Ueno et al. had to be attributed to an unrecognized artefact(s), possibly trapping of very small fragments of the hydrophobic beads with their bound detergent inside the just-reconstituted membrane vesicles. We still think this could have been the case. Nevertheless, we now also think that our previous conclusion that overall dissociation of C_12_E_8_ from the membranes was proceeding with a half time of 300–400 ms [8] has to be revised, too.

We indeed more recently performed new experiments on this issue, but now using a stopped-flow fluorescence assay with brominated detergent analogues. This method is based on the fact that the intrinsic fluorescence of protein tryptophan (Trp) residues located inside the membrane may get quenched by the bromine atoms residing on the hydrophobic chain of such detergent molecules in the vicinity of these Trp residues: the rate of fluorescence quenching can therefore reveal the rate of detergent binding to the membranes, while fluorescence will of course recover if the brominated detergent dissociates from the membrane [9]. In fact, exploiting fluorescence quenching by brominated analogues has previously made it possible to study the interaction of brominated lipids with transmembrane proteins. For instance, brominated lipids was recently used to measure binding constants of lipids to channels [10,11], and even earlier for evaluating their interactions with SERCA1a [12], including in the presence of non-brominated detergent [13]. The SERCA1a from SR native membranes is a particularly appropriate target for such Trp fluorescence quenching studies, since this single polypeptide chain of 994 residues contains 13 tryptophan residues among which 12 are located rather symmetrically in the transmembrane domain [14], mostly at the lipid-protein interface [12,15]. Brominated analogues of two different detergents were used: one derived from octaethylene-glycol-dodecylether (C_12_E_8_) and the other one from the widely used non-ionic detergent dodecyl-maltoside (DDM).

Surprisingly, stopped-flow results with *5*,*6*-*Br*_*2*_C_12_E_8_ were not consistent with the results obtained in 1987 using rapid filtration and ^14^C-labelled C_12_E_8_: we found that upon strong dilution into detergent-free buffer of membranes previously incubated with non-solubilizing concentrations of the brominated *5*,*6*-*Br*_*2*_C_12_E_8_, the recovery of the protein intrinsic fluorescence was very fast (with half times shorter than 10 ms), suggesting that dissociation of this detergent from the membranes is probably much faster than what we deduced originally from the filtration experiments with non-brominated C_12_E_8_ [8].

Yet, when the brominated (and again ^14^C-labelled) analogue of C_12_E_8_ was used in rapid filtration experiments, we obtained results consistent with those previously obtained for the non-brominated compound, with a slow apparent dissociation rate for the different molecules. We then became aware of a previously unsuspected methodological bias in our past and present filtration experiments, a bias arising from the combination of the local crowding of the membranes adsorbed onto the filters with the favourable partition of detergent into these filter-loaded membranes and the limited range of perfusion rates achievable with the rapid filtration equipment. As a consequence, and based on our new data with the brominated analogue, we now suggest that the exit of C_12_E_8_ from the membranes into the water phase is much more rapid than previously envisioned, and indeed occurs with a half time not larger than a few milliseconds.

We also here report the fluorescence quenching and de-quenching experiments performed with another detergent, 5,6-Br_2_DDM. In those experiments, fluorescence changes during detergent binding to or dissociation from SR membranes revealed both fast components, as for 5,6-Br_2_C_12_E_8_, and also much slower components specific for DDM. We then briefly discuss association, dissociation and flip-flop rates for these two detergents.

## Materials and methods

The brominated detergents used in the present study, octaethylene-glycol*-5*,*6-*dibromododecylether (*5*,*6-Br*_*2*_C_12_E_8_, MW = 696 g.mol^-1^) and dibromo-dodecyl-β-D-maltoside (*5*,*6-Br*_*2*_DDM, MW = 668 g.mol^-1^) were synthesized as previously described [9,16], including in ^14^C-labelled form. Non-brominated C_12_E_8_ (MW = 538 g.mol^-1^) was obtained from Nikko and non-brominated DDM (MW = 510 g.mol^-1^) was from Anatrace Inc.. In contrast to non-brominated compound, dry *5*,*6-Br*_*2*_C_12_E_8_ had an oily and yellowish appearance (presumably due to the bromine atoms themselves, since ^1^H NMR spectra confirmed the absence of detectable impurities), but this did not prevent complete solubilization at concentrations as high as 100 mg/ml (like the non-brominated C_12_E_8_). Detergent stock solutions were subsequently prepared in deionised water at concentrations of 100 mM (for instance, 55 mg/ml and 70 mg/mL for the unbrominated and brominated *5*,*6-Br*_*2*_C_12_E_8_, respectively), together with more dilute solutions (e.g. 5.5 mM for C_12_E_8_, 10 mM for DDM), and more concentrated ones if desired (e.g. 100 mg/mL C_12_E_8_, i.e. 186 mM, or 200 mg/ml DDM i.e. 390 mM). These nominal values (±10%), based on weight measurements, were in agreement with independent estimates based on measurement of the polyethyleneglycol contents of the detergent *via* its reaction with ammonium cobalto-thiocyanate and extraction into ethylene dichloride [17–19].

The biological membranes used here were sarcoplasmic reticulum (SR) vesicles extracted from rabbit fast twitch muscle (and containing about 0.5 g lipids/g membrane proteins), as in [8] and [20]. SR vesicles were prepared during the period from 2006 to 2009 and stored at -80°C until being used, under conditions that do not alter their biological properties, i.e. in the presence of 0.3 M sucrose. Three different SR preps were used for the present experiments, which were performed over several years, with no loss of ATPase activity observed on this period. Membrane preparation from the rabbit was carried out in strict accordance with the recommendations and after agreement from the Ethic committee of the “Commissariat à l’Énergie Atomique et aux Énergies Alternatives” (CEA agreement #E 91 272 106; see S1 NC3Rs ARRIVE Guidelines Checklist). All surgery was performed after killing rabbit by bleeding after a blow to the neck with a metal bar, as quickly as possible to minimize suffering (for a detailed procedure of subsequent steps, see [21]). The concentration of SR membranes in each experiment is expressed in terms of their protein contents, *i*.*e*. in μg of protein/mL. In all experiments the buffer contained 100 mM KCl, 1 mM MgCl_2_ and 50 mM Tes-Tris at pH 7.5 and 20°C (designated as “pH 7.5 buffer”), together with 0.05 mM free Ca^2+^ (0.1 mM total Ca^2+^ and 0.05 mM EGTA) to optimize preservation of SERCA1a, the main protein of the SR membranes.

For each detergent, its critical micellar concentration (cmc) at pH 7.5 was estimated using 40 μM methyl orange, as previously described [22,23]. Light scattering (at 290 nm) and Trp fluorescence (λ_ex_ and λ_em_ at 290 nm and 340 nm, respectively) of the membranes, in the absence or presence of detergent, were measured as previously described (see e.g. [15]), and sometimes simultaneously, using a Spex Fluorolog equipped with two independent monochromators in the “T” configuration. In some cases also, light scattering and Trp fluorescence changes were measured during slow continuous dilution inside the cuvette of the contents of a detergent-containing mechanically-driven syringe. In those cases, detergent delivery from the syringe (containing a 5.5 mM C_12_E_8_ or a 10 mM DDM solution) into the 2 ml spectrophotometer cuvette was performed at 400 μl/hour, resulting within half an hour in a final addition of 0.5 mM or 0.9 mM detergent together with an up to 10% dilution of the membranes. Note that this detergent delivery procedure not only makes it possible to collect a large number of data points, but also minimizes the artefactual transient solubilization of membranes which might occur when a droplet of concentrated detergent is added from an ordinary pipet [24].

Stopped-flow experiments were performed using a Biologic SFM 3 equipment (see e.g. [25]), but here with mixing in different volume-to-volume ratios of the contents of the two syringes. The nominal dead-time of the machine is about 3 ms. The excitation wavelength was 290 nm, and fluorescence emitted at 340 nm was detected using a combination of filters (MTO J324 + A340).

Rapid filtration measurements were performed using a Biologic equipment, as in [8], and Whatman GF/F glass fiber filters. Such filters have pore diameters larger than the typical diameter (0.06–0.3 μm) of SR vesicles, and therefore retain the SR membranes thanks to adsorption of these membranes onto the pore walls. The total volume of “wetting fluid” in such filters is ~100 μL, but membranes are loaded onto the filter using a funnel of diameter smaller than the one of the filter, and they probably adsorb mainly onto the walls of the central pores of the filter, say, within ~50 μL fluid. The diameter of the syringe delivering the perfusion fluid is intermediate. Perfusion rates were 2–4.5 ml/s (faster rates for shorter periods).

**Fig 1:**
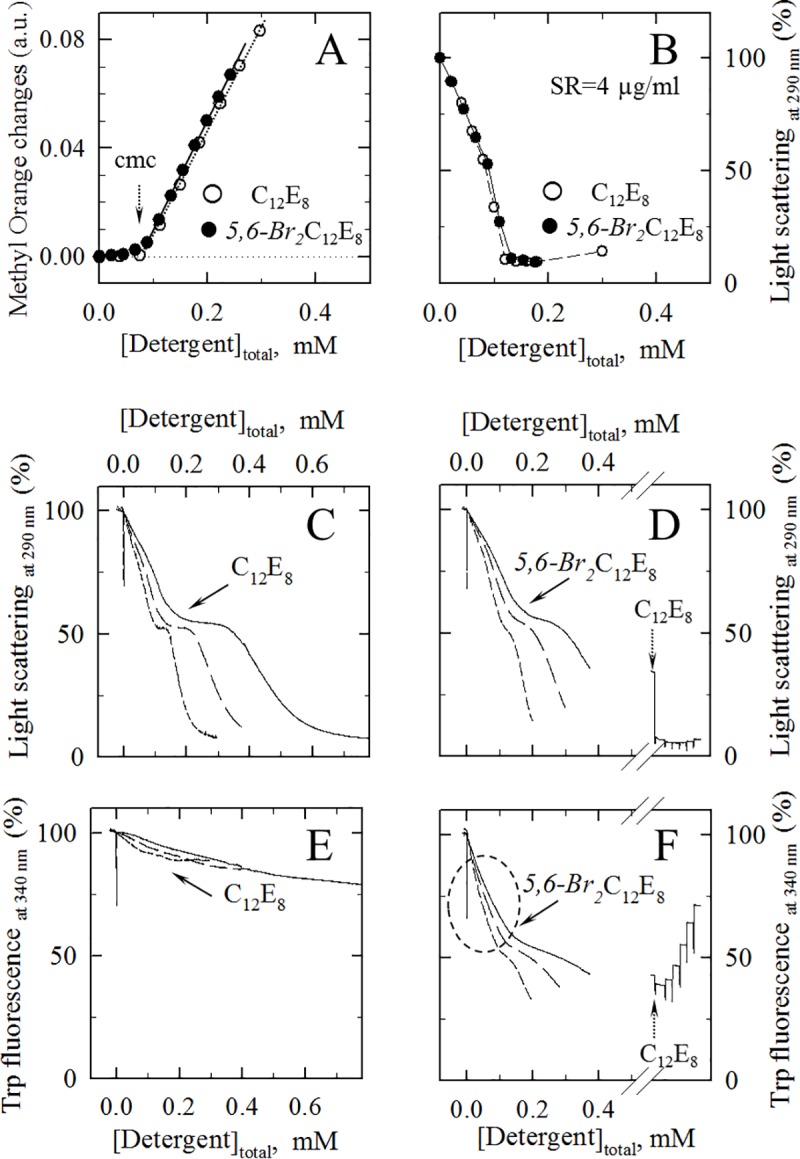
Comparison of C_12_E_8_ and *5*,*6-Br*_*2*_C_12_E_8_ properties: Critical micellar concentration, steady-state interaction with SR vesicles as deduced from light scattering, and effects on protein intrinsic fluorescence. (A) Detergent cmc (arrow) for C_12_E_8_ and *5*,*6-Br*_*2*_C_12_E_8_, as determined from the spectral changes of 40 μM methyl orange (expressed as ΔA_415 nm_− ΔA_500 nm_, in absorbance units) in the presence of increasing concentrations of these detergents. (B) Perturbation by C_12_E_8_ and *5*,*6-Br*_*2*_C_12_E_8_ of the 90° light scattering (at 290 nm) by SR vesicles (at 4 μg protein/ml). In Panels A and B, closed symbols correspond to *5*,*6-Br*_*2*_C_12_E_8_, open symbols correspond to non-brominated C_12_E_8_. (C and D) Perturbation by C_12_E_8_ (C) and *5*,*6-Br*_*2*_C_12_E_8_ (D) of light scattering by SR vesicles, as in Panel B, but here recorded upon continuous delivery of concentrated detergent from a small syringe into the spectrophotometer cuvette, and in the presence of different concentrations of membranes (20, 50 or 100 μg/ml of protein, short dash, long dash, and continuous lines, respectively). Recorded signals were not corrected for the resulting small dilution of membranes (10% at 0.5 mM detergent). The few data points for “negative” detergent concentrations correspond to data recorded before actuation of the syringe. (E and F) Perturbation by C_12_E_8_ (E) and *5*,*6-Br*_*2*_C_12_E_8_ (F) of the intrinsic fluorescence signal for SR vesicles under the above conditions (continuous delivery of detergent and in the presence of different concentrations of membranes, again at 20, 50 or 100 μg/ml of protein). In Panels D and F, after addition of *Br*_*2*_C_12_E_8_ up to 0.44 mM to the 100 μg/ml SR suspension, increasing amounts of non-brominated C_12_E_8_ were finally added to the ~2 ml suspension, up to about 6 mM (2, 2, 4, 8, 16, and finally 32 μl of a very concentrated solution of C_12_E_8_−100 mg/ml, *i*.*e*. 186 mM). Blank values (buffer only, in the absence of membranes) were subtracted from the “fluorescence” signal, but detergent-induced dilution and photolysis were not corrected for.

**Fig 2:**
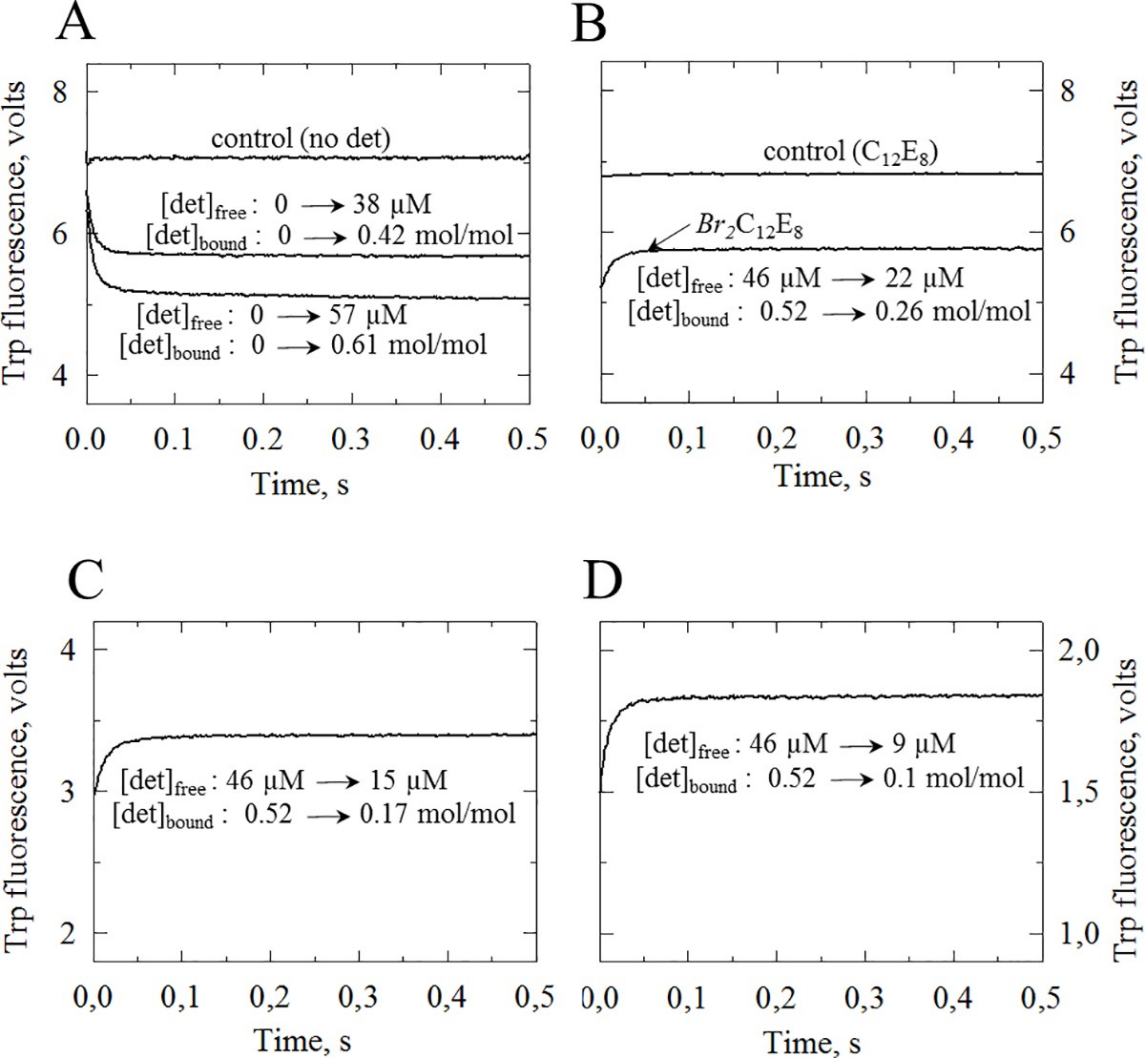
**Kinetics of Trp fluorescence quenching or recovery, observed in stopped-flow experiments upon binding (A) or dissociation (B, C, D) of *5*,*6-Br*_*2*_C_12_E_8_ to or from sarcoplasmic reticulum membranes.** (A) *Binding experiments*. For each shot in these experiments, 36 μl of a suspension of SR membranes at 440 μg protein/ml was mixed with 164 μl (i.e. at a ratio of ~1:4.5 vol:vol) of *5*,*6-Br*_*2*_C_12_E_8_ at zero (top trace, “control”), 73 μM (intermediate trace) or 110 μM (bottom trace) total concentrations, resulting after mixing in final concentrations of 80 μg protein/ml and zero, ~60 μM or ~90 μM of final *total* detergent, respectively (*i*.*e*. 0, ~38 μM or ~57 μM of *free* detergent and 0, ~22 μM or ~33 μM of *bound* detergent, *i*.*e*. 0, ~0.42 or ~0.61 mol bound detergent/mol membrane lipid, as estimated on the basis of the well-established binding characteristics for the non-brominated C_12_E_8_ [4,8] and the fairly similar properties for *5*,*6-Br*_*2*_C_12_E_8_, see Methods. (B, C, D) *Dissociation experiments*. In all cases, a suspension of SR membranes at 440 μg protein/ml was first preincubated for a few minutes with *5*,*6-Br*_*2*_C_12_E_8_ at a total concentration of 200 μM, out of which the *free* detergent concentration is only 46 μM, a non-solubilizing concentration (see Fig 1), and bound *5*,*6-Br*_*2*_C_12_E_8_ is ~154 μM, *i*.*e*. ~0.52 mol bound detergent/mol membrane lipid. For Panel B experiment, this suspension was then mixed with buffer alone in the stopped-flow machine (lower trace), at the same vol:vol ratio (36 μl + 164 μl, *i*.*e*. ~1:4.5) as in the binding experiments illustrated in Panel A (hence the similar Y-axis scales). A control was also included (upper trace in Panel B), in which non-brominated C_12_E_8_ at the same concentration was used, instead of *5*,*6-Br*_*2*_C_12_E_8_. For Panel C experiment, a larger dilution factor was used, (20 μl + 180 μl, *i*.*e*. 1:9), so that the final concentration of membranes was smaller than for Panel B, hence the smaller Trp fluorescence signal (see Y-axis scales). For Panel D experiment, an even larger dilution factor was used (10 μl + 180 μl, *i*.*e*. 1:18). Final free and bound detergent concentrations after reaching equilibrium in the various situations are ~22 μM and 14 μM, ~15 μM and 5 μM, or ~9 μM and 1.5 μM (i.e., 0.26, 0.17 or 0.10 mol bound detergent/mol membrane lipid, respectively), estimated as above-mentioned. Note that similar dissociation experiments, repeated now starting from membranes preincubated with a lower total concentration of *5*,*6-Br*_*2*_C_12_E_8_ (100 μM instead of 200 μM), also led to qualitatively similar recordings (but of course less quenched initial and final fluorescence levels). Traces were usually recorded with 1–2 ms electronic filtering, and correspond to the average of 5–8 shots; in all cases, the “blank” optical signal (about 0.4 volts) measured in the total absence of membranes has been subtracted from the recorded signal.

Remember that in the presence of a low, non-solubilizing (*i*.*e*. only “perturbing” [4,26]) concentration of detergent, the total concentration of this detergent is equal to the sum of its free concentration (in the water phase) and its bound concentration (inserted in the membranes, but here expressed per ml of water phase), the latter, at a given free detergent concentration, being dependent on the amount of membranes [27]). The detergent binding isotherms used here, for planning and roughly estimating free and bound concentrations of the brominated detergents under the various situations explored in Figs 2 and 5, were deduced (see *e*.*g*. [27,28]) from the membrane concentration-dependent shifts in the detergent-dependent light scattering curves of Figs 1 and 4. They were similar for both versions of each detergents, and consistent with the binding isotherms already published for the non-brominated versions of either C_12_E_8_, [4,8] or DDM [29].

## Results

Brominated detergents have previously been described as useful tools to study detergent-membrane interaction, thanks to their fluorescence quenching properties [9]. In the present experiments (Fig 1), we first ascertained that brominated *5*,*6-Br*_*2*_C_12_E_8_ detergent had physico-chemical properties very similar to those of the parent detergent, C_12_E_8_. Indeed we found a fairly similar cmc for the brominated or unbrominated detergent, ~75 μM (arrows in Fig 1A), as tested with methyl orange [22,23], and fairly similar abilities to perturb light scattering by sarcoplasmic reticulum (SR) membrane vesicles (Fig 1B). At low, non-solubilizing concentrations, such light scattering changes may reflect changes in membrane shape as well as changes in the refraction index of the membrane vesicles, while at higher concentrations, they mainly reflect solubilization of the membranes down to poorly scattering detergent/protein/lipids mixed micelles, as previously described for other non-brominated detergents [4,13,26]. Furthermore, we observed similar abilities of the brominated and the non-brominated detergents to partition into the membranes, as determined from the fact that at increasingly higher concentrations of membranes, light scattering curves were shifted to the right (see e.g. [9,28]) in a quantitatively fairly similar manner (see Fig 1C for C_12_E_8_ and Fig 1D for *5*,*6-Br*_*2*_C_12_E_8_). At low, non-solubilizing as well as high concentrations, brominated detergent was also exhibited the typical influence of C_12_E_8_ on the enzymatic activity of the Ca^2+^-dependent ATPase SERCA1a, the main protein present in these membranes [4,13].

Of major importance, the brominated *5*,*6-Br*_*2*_C_12_E_8_ was able to quench the intrinsic fluorescence of the Trp residues of the proteins in these membranes in a detergent- and membrane concentration-dependent manner (Fig 1F). This quenching can be assigned to contact or very short-distance fluorescence quenching by the bromine heavy atoms [12,20] located on the hydrophobic chain of this detergent, as previously observed for brominated phospholipids [9,12,13] as well as for other brominated detergents like dibromo-dodecylmaltoside [9,20]. At the intermediate plateau level, corresponding to saturation of membranes by detergent (just before their solubilisation), the extent of fluorescence quenching by the membrane-bound brominated detergent was already very significant (close to 40–50%), presumably because in SERCA1a, most Trp residues are embedded in the transmembrane region, and located very close to its hydrophobic surface [14]. Fluorescence quenching was further increased at solubilizing detergent concentrations, and such quenching could be reversed by addition of excess non-brominated C_12_E_8_ at the end of the experiment, as expected (Fig 1F, right side). In contrast, non-brominated C_12_E_8_ only marginally interfered with the Trp intrinsic fluorescence (Fig 1E): the minor decrease in signal illustrated in Fig 1E mainly corresponds to detergent-induced dilution and time-dependent photolysis, which have not been corrected for in Fig 1E & 1F. It should be noted that when detergent was added to the membranes at non-solubilizing concentrations, it nevertheless interacted with both sides of the membrane on the time-scale of the present experiments, since transmembrane flip-flop of C_12_E_8_ has been shown to be faster than 300–400 ms [8].

The contact and dose-dependent quenching exerted by *5*,*6-Br*_*2*_C_12_E_8_ on the fluorescence of membrane-embedded Trp residues made it possible to take advantage of stopped-flow fluorescence for measuring the kinetics of *5*,*6-Br*_*2*_C_12_E_8_ binding to, or dissociation from, these membranes. Binding experiments were the first ones to be performed, and results are illustrated in Fig 2A. As a preliminary control, SR membranes were first mixed with buffer alone, to reveal the Trp fluorescence reference level in the absence of brominated detergent (about 7 volts, top trace in panel A). SR membranes were then mixed with two non-solubilizing concentrations of *5*,*6-Br*_*2*_C_12_E_8_, resulting in free detergent concentrations of 38 μM (intermediate trace in panel A) and 57 μM (bottom trace in panel A), respectively (see figure legends for details). In both cases, the Trp fluorescence level dropped rapidly, with observed half times shorter than 10 ms. Note that the recorded traces begin (at t = 0) significantly below the control level of 7 V, presumably because the initial fluorescence drop occurred within the dead time of the stopped-flow equipment (a few ms), and this makes it impossible to more precisely estimate the true rate constants of the fluorescence changes in Panel A. However, it is clear that *5*,*6-Br*_*2*_C_12_E_8_ insertion into the membranes is very fast, even at the non-solubilizing concentrations used here. Recording the traces over longer periods did not reveal anything except the expected photolysis.

In a second step, dissociation experiments were performed, in which SR membranes pre-incubated with *5*,*6-Br*_*2*_C_12_E_8_ were mixed with detergent-free buffer, with significant dilution. For the lower trace illustrated in panel B of Fig 2, concentrated SR membranes, pre-incubated with a relatively high but nevertheless non-solubilizing concentration of *5*,*6-Br*_*2*_C_12_E_8_ (200 μM total, *i*.*e*. 46 μM free, a concentration lower than both the cmc and the critical solubilisation concentration (csc) of C_12_E_8_ [30]), were mixed with buffer alone, at a ratio of 1:4.5 v:v as for the binding experiments in Panel A. Fluorescence recovery was completed after a few tens of milliseconds only. At the end of the recording, the fluorescence level still remained below that of the control experiment in Panel A, in agreement with the fact that upon reaching the final equilibrium, the free detergent concentration only drops to about 22 μM, not to zero. A second control for this dissociation experiment was performed using non-brominated C_12_E_8_ instead of *5*,*6-Br*_*2*_C_12_E_8_. In that case, fluorescence remained at the control level (Fig 2B, upper trace).

To explore other conditions, experiments with *5*,*6-Br*_*2*_C_12_E_8_ were repeated at higher dilution ratios, either 1:9 v:v (Fig 2C) or 1:18 v:v (Fig 2D), and in both cases starting from the same concentrations of *5*,*6-Br*_*2*_C_12_E_8_ in the pre-incubated concentrated sample of 200 μM (initial free *Br*_*2*_C_12_E_8_ of about 46 μM, as in Fig 2B). Initial fluorescence levels immediately after mixing and final levels after reaching equilibrium were different, but in all cases, including at the highest dilution and therefore lowest free detergent concentrations, the kinetics of fluorescence recovery remained very fast, with observed half times shorter than 10 ms and no indication of any slower phase. In particular, we found no evidence for any recovery phase with the 300–400 ms half time which had been observed in the earlier rapid filtration experiments [8].

**Fig 3:**
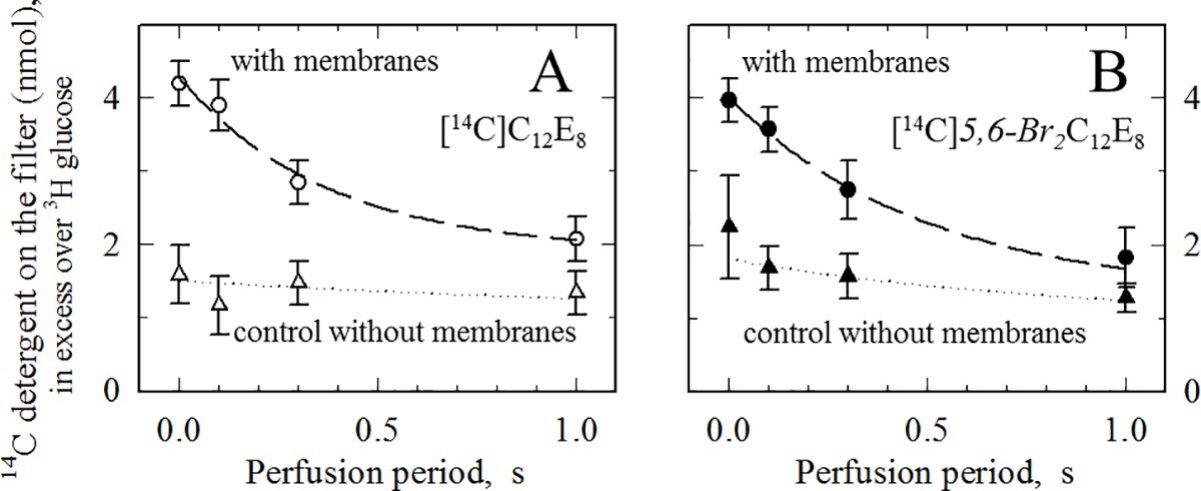
**Rapid filtration experiments with either ^14^C-labelled C_12_E_8_ (A) or ^14^C-labelled *5*,*6-Br*_*2*_C_12_E_8_ (B).** SR membranes at 80 μg/ml (circles) were pre-equilibrated with a total concentration of 10 μM of either [^14^C]C_12_E_8_ (A) or [^14^C]5,6-*Br*_*2*_C_12_E_8_ (B) (at about 10^6^ cpm/μmol) in buffer also containing 10 μM [^3^H]glucose. 1 ml was loaded on a glass fiber filter and perfused for various periods with detergent-free and membrane-free buffer (having unlabelled detergent in the perfusion buffer, at the same low concentration as in the initial membrane suspension, made little difference, not shown). The filter was then counted (circles). As in [8], [^3^H] counts allowed to compute the volume of initial fluid remaining on the filter, in particular for the time zero point, for which no perfusion took place (the Y axis is labelled as “[^14^C]detergent in excess over [^3^H]glucose”). The average of 2–7 points is shown. Control experiments were repeated in the absence of SR membranes (triangles). In these control experiments, disappearance from the filter of the merely “adsorbed” detergent molecules was slower than in the 1987 experiments (squares in Fig 5A of ref. [8]): this slower desorption correlates with our using here a concentration of detergent, 10 μM, significantly lower than the one used in 1987 (41 μg/ml, i.e. about 75 μM), with the aim of unambiguously keeping the detergent concentration in the range of non-solubilizing concentrations, but with the side consequence that the adsorbed detergent molecules possibly experience slower dissociation kinetics. The exchange dynamics of adsorbed ligands has already in some cases been suggested to be dependent on the amount of ligand adsorbed (e.g. [31]).

We then wondered whether the presence of bromine atoms in the detergent could be responsible for the discrepancy between the rapid fluorescence recovery observed in the above stopped-flow fluorescence experiments upon *Br*_*2*_C_12_E_8_ dissociation (Fig 2B–2D) and the much slower loss of ^14^C-labelled C_12_E_8_ which we had observed in the 1987 rapid filtration experiments [8]. Importantly, this was *not* the case (Fig 3), because when we repeated rapid filtration experiments, in a similar way as in 1987 but now using either ^14^C-labelled C_12_E_8_ or ^14^C-labelled *Br*_*2*_C_12_E_8_, we obtained very similar results in both cases: half times for the apparent loss of these ^14^C-detergents from the membranes were in the 300–500 ms range (Fig 3A and 3B) both for the non-brominated and for the brominated detergent, as previously found for the non-brominated compound [8]. The above-mentioned discrepancy therefore does *not* arise from the presence or absence of bromine atoms on the detergent, but from the methods used to follow their dissociation kinetics.

**Fig 4:**
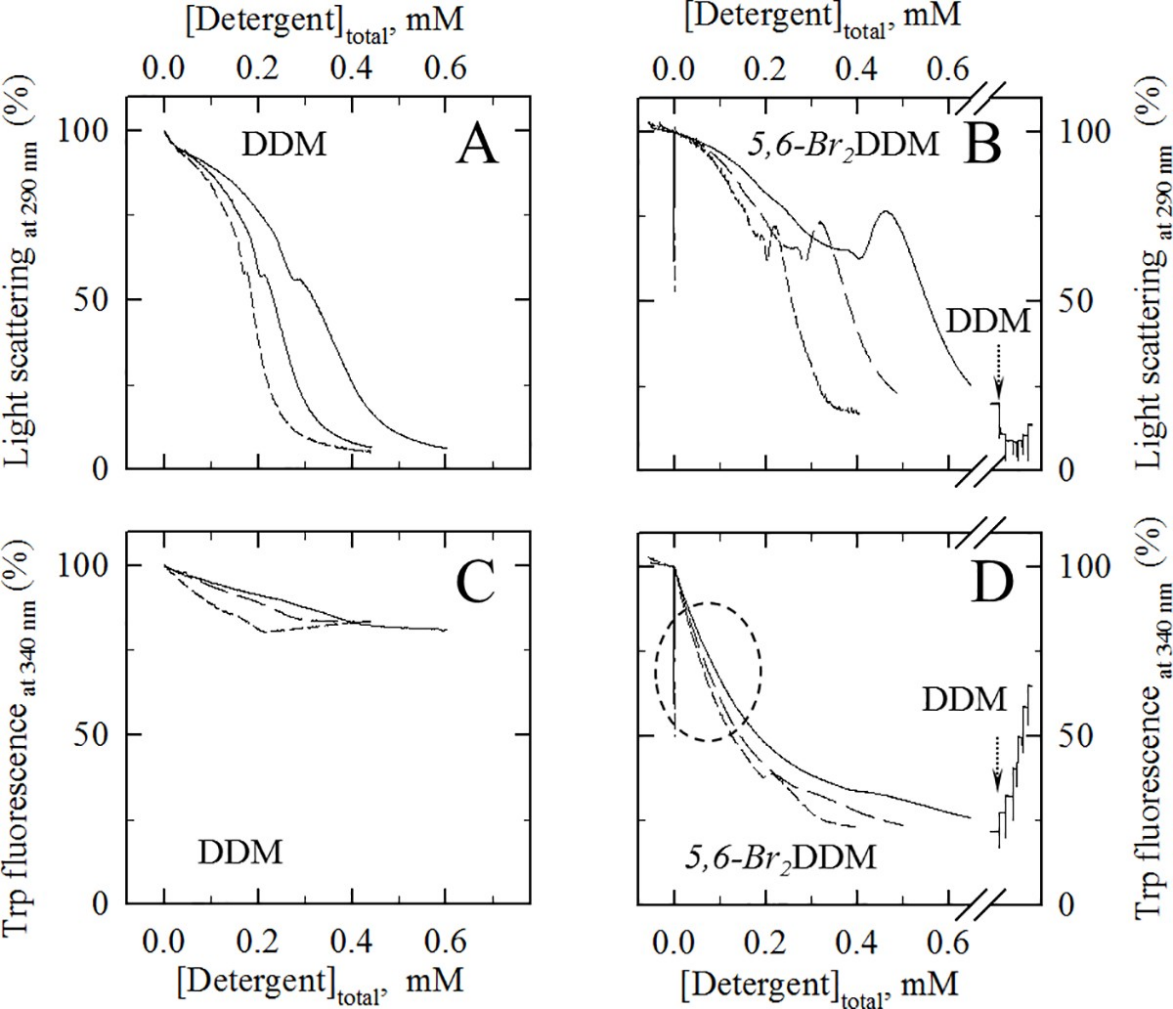
Interaction of either DDM or *5*,*6-Br*_*2*_DDM with SR vesicles at various concentrations: light scattering and Trp fluorescence changes. (A and B) Perturbation by DDM (A) and *5*,*6-Br*_*2*_DDM (B) of light scattering by SR vesicles, here recorded upon continuous delivery of concentrated detergent from a small syringe into the spectrophotometer cuvette and in the presence of different concentrations of membranes (20, 50 or 100 μg/ml of protein), as for Fig 1C–1F. Recorded signals were not corrected for the resulting small dilution of membranes (10% at 0.9 mM detergent). The few data points for “negative” detergent concentrations correspond to data recorded before actuation of the syringe. (C and D) Perturbation by DDM (C) and *5*,*6-Br*_*2*_DDM (D) of the intrinsic fluorescence signal for SR vesicles under the above conditions (continuous delivery of detergent and in the presence of different concentrations of membranes, again at 20, 50 or 100 μg/ml of protein). In Panels B and D, after addition of *Br*_*2*_DDM up to 0.8 mM to the 100 μg/ml SR suspension, increasing amounts of non-brominated DDM were finally added to the ~2 ml suspension, up to about 12 mM (2, 2, 4, 8, 16, and finally 32 μl of a very concentrated solution of DDM -200 mg/ml, *i*.*e*. 390 mM). Blank values (buffer only, in the absence of membranes) were subtracted from the “fluorescence” signal, but detergent-induced dilution and photolysis were not corrected for.

At this stage, we also investigated another brominated detergent, *5*,*6-Br*_*2*_DDM. Just like *5*,*6-Br*_*2*_C_12_E_8_, *5*,*6-Br*_*2*_DDM has properties similar to those of its non-brominated parent, DDM. The very similar cmc (0.14–0.16 mM) and solubilisation efficiency of *5*,*6-Br*_*2*_DDM and DDM have recently been published, together with their similar perturbing effects on ATPase activity (see supplemental information in [20]). Observed effects of the two versions of DDM on light scattering by SR, shown in Fig 4A and 4B are rather similar, although *5*,*6-Br*_*2*_DDM has a more marked tendency to induce a transient increase in light scattering at intermediate concentrations of detergent. Note that such an increase has already been observed for a number of detergents in the region where their binding to membranes, and more especially to pure liposomes, becomes cooperative and solubilisation starts. It is probably due to macroscopic reorganization and transient aggregation of the detergent-saturated membranes, *e*.*g*. opening and fusion of the vesicles [24,30]. As anticipated from the present results obtained with *5*,*6-Br*_*2*_C12E8 (Fig 1D) or those obtained previously with other brominated analogues of DDM [9], *5*,*6-Br*_*2*_DDM was also able to quench the fluorescence of SR Trp residues (Fig 4D) even before membrane solubilisation. This quenching is even larger than in the case of *5*,*6-Br*_*2*_C_12_E_8_ (compare Fig 4D vs Fig 1F).

**Fig 5:**
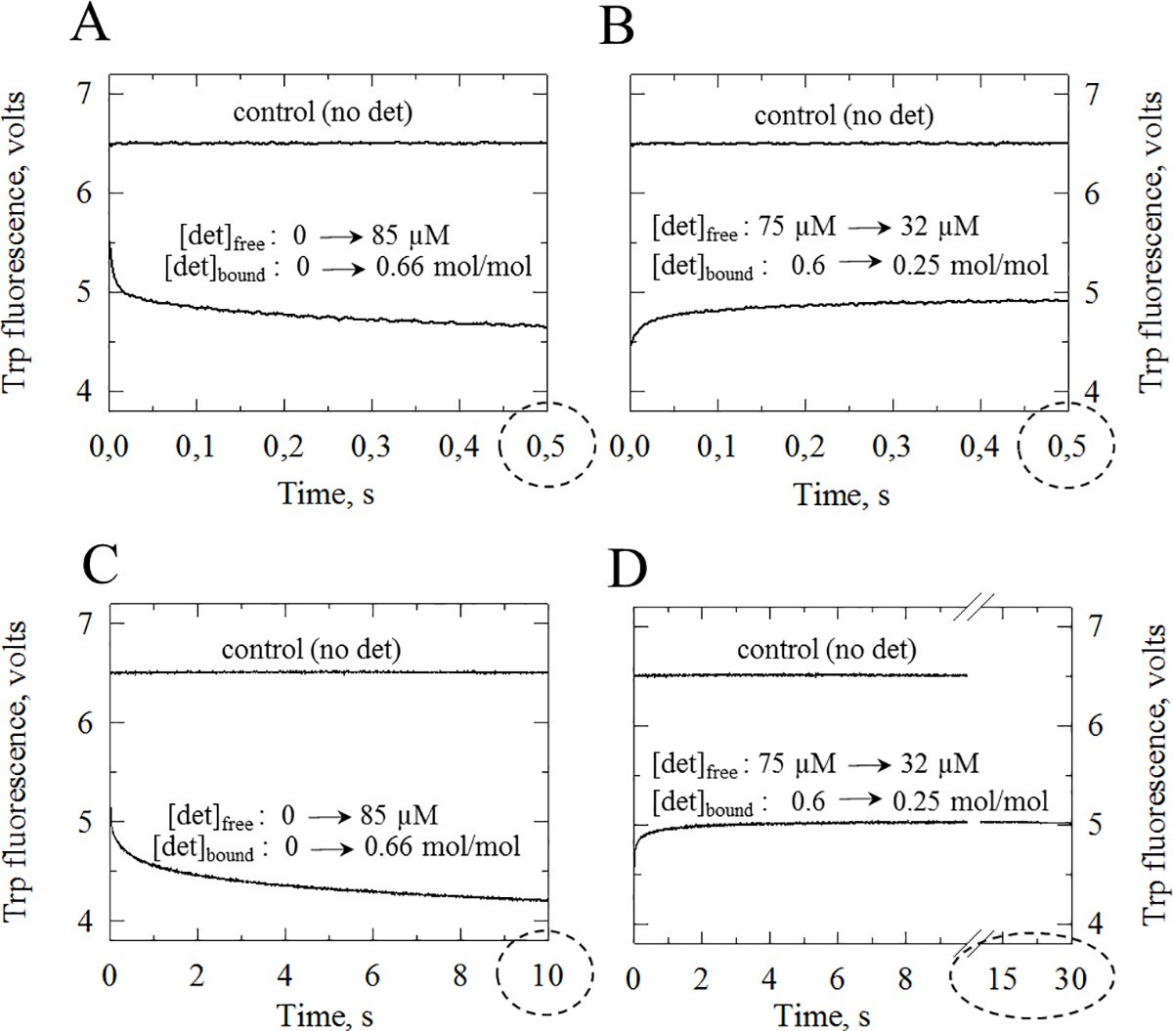
**Kinetics of Trp fluorescence quenching or recovery, observed in stopped-flow experiments upon binding (A, C) or dissociation (B, D) of *5*,*6-Br*_*2*_DDM to or from sarcoplasmic reticulum membranes.** (A, C) *Binding experiments*. For each shot in these experiments, 36 μl of a suspension of SR membranes at 440 μg protein/ml was mixed with 164 μl (i.e. at a ratio of ~1:4.5 vol:vol) of *5*,*6-Br*_*2*_DDM at zero (control top trace) or 145 μM (bottom trace) total concentration, resulting after mixing in final concentrations of 80 μg protein/ml and zero or ~120 μM of final *total* detergent, respectively, and therefore, as estimated from the results in Fig 4 (and [29]), in 0 or ~85 μM of *free* detergent (and 0 or 35 μM of *bound* detergent, *i*.*e*. 0 or 0.66 mol bound detergent/mol membrane lipid). The signal was recorded over 10 s out of which the first 0.5 s are shown in panel A, while the entire trace is shown in Panel C. (B,D) *Dissociation experiments*. A suspension of SR membranes at 440 μg protein/ml was first preincubated with *5*,*6-Br*_*2*_DDM at a total concentration of 250 μM, out of which, as described above, the *free* detergent concentration is estimated to be around 75 μM (a non-solubilizing concentration) and bound *5*,*6-Br*_*2*_DDM to be around 175 μM, i.e. 0.6 mol bound detergent/mol membrane lipid. This suspension was then mixed (and diluted) with buffer alone in the stopped-flow machine (lower trace), at the same vol:vol ratio (36 μl + 164 μl, *i*.*e*. ~1:4.5) as in the binding experiments above (hence the similar Y-axis scales). The signal was recorded over 30 s out of which the first 0.5 s are shown in panel B, while the entire trace is shown in Panel D. The above control without detergent is also reproduced here (upper traces in all panels).

Stopped flow experiments were then performed to reveal the kinetics of *5*,*6-Br*_*2*_DDM binding or dissociation. The kinetics observed for *5*,*6-Br*_*2*_DDM binding or dissociation again comprised a very rapid phase, within milliseconds, poorly resolved by our stopped-flow equipment. While signals were stable after 100 ms for *5*,*6-Br*_*2*_C_12_E_8_ (Fig 2B–2D), the kinetics for *5*,*6-Br*_*2*_DDM binding or dissociation also comprised a slower phase, observable both over 0.5 s (Fig 5A and 5B) and still observed if signal recording is extended over a few seconds (see Fig 5C and 5D). Over such a longer period of time, the slow component in the dissociation trace (Fig 5D) was still present but less obvious than in the association trace (Fig 5C). A possible reason for this particular behaviour will be discussed later.

## Discussion

One of the main conclusions drawn from this work is that caution needs to be taken when designing a method for measuring the rate of dissociation of detergent from membranes. Our first attempt in 1987 using a rapid filtration method had suggested for C_12_E_8_ a half time for dissociation in the 300–400 ms range, both from egg phosphatidylcholine unilamellar or multilamellar vesicles and from protein-containing sarcoplasmic reticulum (SR) membrane vesicles (Fig 5 in [8]). But stopped-flow experiments using 5,6-*Br*_*2*_C_12_E_8_ now reveal a much shorter half time (a few milliseconds), hardly distinguishable from the dead time of the stopped-flow equipment itself (Fig 2B–2D of the present paper), despite the fact that [^14^C]*5*,*6*-*Br*_*2*_C_12_E_8_ behaves like [^14^C]C_12_E_8_ in rapid filtration experiments (Fig 3 of the present paper). We propose a tentative explanation for this discrepancy below.

But before doing so, it is perhaps worth to address a conceivable objection to our fluorescence recovery experiments performed with *5*,*6-Br*_*2*_C_12_E_8_: as it is only the fluorescence of Trp residues which is monitored in these experiments, it might be argued that such stopped-flow experiments only reveal the possibly fast rate of dissociation of those detergent molecules which are in contact with the proteins, and not the dissociation rate of bulk detergent molecules present elsewhere in the lipid phase and possibly located too far from the protein Trp residues to be efficient quenchers. We in fact consider this possibility as highly unlikely, for three reasons: (1), detergent molecules in contact with membrane proteins probably exchange position very rapidly with the “bulk” detergent molecules, so that on the millisecond time scale it is a homogenous population; in fact, even phospholipid molecules rapidly exchange positions: lipids which were originally described as “immobilized” when they were first studied by electron spin resonance techniques with sub-microsecond timescale sensitivity, were subsequently found to be indistinguishable from “bulk” lipids when they were examined by nuclear magnetic resonance techniques (especially deuterium NMR) with millisecond time scale sensitivity (*e*.*g*. [32,33]); (2), for exerting significant fluorescence quenching on the SERCA1a Trp residues, most of which are located within the membrane [14], *5*,*6-Br*_*2*_C_12_E_8_ molecules must have their aliphatic chains with the bromine atoms correctly inserted in the membrane phase close to the protein hydrophobic surface, and it is difficult to envisage how such *5*,*6-Br*_*2*_C_12_E_8_ molecules could be more easily exchangeable than bulk detergent molecules simply embedded within the membrane lipids; (3), stopped-flow fluorescence binding and dissociation experiments similar to the ones reported for *5*,*6-Br*_*2*_C_12_E_8_ in Fig 2, but performed with another non-ionic brominated detergent, *5*,*6-Br*_*2*_DDM, reveal for both binding and dissociation the existence of much slower components of fluorescence changes (Fig 5, see further discussion below) than for *5*,*6-Br*_*2*_C_12_E_8_. Similar slow components were also mentioned with another brominated version of DDM, *7*,*8-Br*_*2*_DDM [9] and they had been considered to reflect the previously suggested slow flip-flop of DDM compared with C_12_E_8_ [30]. The conclusion of such an observation with *Br*_*2*_DDM necessarily is that using brominated detergents and a fluorescence quenching or de-quenching assay cannot *per se* be responsible for the absence of slow events during dissociation of *5*,*6-Br*_*2*_ C_12_E_8_. So the rapid kinetics illustrated in Fig 2B–2D probably do reflect rapid overall dissociation of *5*,*6-Br*_*2*_C_12_E_8_ from the membrane while a different detergent, *e*.*g*. *5*,*6-Br*_*2*_DDM, may have different properties regarding its kinetics of overall interaction with the membrane and in particular its kinetics of transmembrane flip-flop (see further discussion below).

Back to the methodological issue raised by comparing the very fast events observed in *5*,*6-Br*_*2*_C_12_E_8_ fluorescence de-quenching dissociation experiments and the seemingly much slower dissociation of ^14^C-labelled *5*,*6-Br*_*2*_C_12_E_8_ (or C_12_E_8_) in rapid filtration experiments, we now think that the rapid filtration experiments with ^14^C-labelled versions of C_12_E_8_ give artefactual results. In such filtration experiments, the local concentration of the membrane vesicles adsorbed onto the glass fiber filter is very high. Consequently, the resulting partition of detergent, here C_12_E_8_, between water and the membranes is very much in favour of the bound detergent. For instance, with either 80 or 100 μg protein loaded onto each filter (as in the present Fig 3 experiments, or those in the Fig 5 experiments of [8]), this ratio is ~1/12 or ~1/15 (mol free in water/mol bound to membrane), as calculated using the same detergent binding isotherm for C_12_E_8_ as the one in Fig 2 of [8] and estimating the fluid volume around the filter-loaded membranes to about 50 μL as mentioned under Materials and Methods. Therefore, even if the volume of buffer perfused within a certain period seems to be enough to efficiently rinse the pore contents (e.g. if the filter is perfused with buffer during a 300 ms period at a perfusion rate of 3.3 ml/s, leading to a perfused volume of 1 mL), the perfused buffer will efficiently wash out only the fraction of detergent which is free, while the bound detergent, even if assumed in relatively rapid equilibrium with the free one, will only drop moderately. Therefore, the total amount of detergent on the filter will decrease unduly slowly, with a rate of no significance for the possibly very fast « true » rate of detergent dissociation from membrane. The same artefact will also show up in detergent binding experiments. In 1987, binding of non-brominated C_12_E_8_ to SR membranes measured by rapid filtration, was found to be seemingly slow (Fig 6 in our previous work [8]), but the perfusion rates provided by the filtration equipment were in fact too slow to reveal the fast binding now detected (Fig 2A) by stop-flow fluorescence quenching upon binding of brominated C_12_E_8_. Ironically, the change in intrinsic fluorescence occurring upon binding of non-brominated C_12_E_8_ to SR membranes had already been found to be very fast in 1987 (Fig 6C in our previous work [8]), but this observation was unfortunately misinterpreted. Avoiding these perfusion rate artefacts would require to reduce considerably the amount of membranes loaded onto the filter, but such reduction would be incompatible with keeping a fair signal over noise ratio. Binding of detergent to the filter, in the absence of membranes, is indeed quite significant, even though glass fiber filters are more favourable than other types of filter from the point of view of this non-specific binding. Note that non-specific adsorption is a common problem for brominated versions and radioactively-labelled versions of detergents (either DDM or C12E8): such detergents easily adsorb onto many surfaces (not only to our glass fiber filters, but also various materials, nitrocellulose, Falcon tubes, etc …), even at low concentrations, and with specific kinetics: such adsorption processes of course complicate precise comparisons between different experiments (and particularly, in our stopped-flow fluorescence experiments, comparison of amplitudes). In contrast, in previously published rapid filtration experiments studying dissociation of ^45^Ca initially bound to SERCA1a, potential artefacts due to limited perfusion rates and local crowding did not show up, because ^45^Ca dissociation was measured in the presence, in the perfusion buffer, of either an excess of a strong chelator (EGTA), or an excess of non-radioactive calcium (^40^Ca) [34]. In such experiments, the nitrocellulose filters used do not adsorb calcium, so that calcium binding is restricted to the SR membrane whereas detergents can bind to the entire surface of the filter.

At this point, it is perhaps time to enlarge our discussion and tentatively discuss in more detail the various steps which may influence the observed overall signals in our fluorescence quenching and de-quenching experiments, namely: (i) the detergent movements between the water phase and the external monolayer of the membrane whose kinetics are governed by rate constants k_ass_ and k_diss_, respectively. For simplicity we will neglect the binding and dissociation events occurring on the internal side of the vesicles, since they probably affect an only small fraction of the signal; (ii) the detergent movements from one monolayer of the membrane to the other are governed by rate constants k_flip_ and k_flop_. For simplicity we will assume that these two rate constants are equal. The exact numbers and quantum yields of Trp residues within each monolayer, as well as and their exact degree of accessibility to detergent, will of course also influence the amplitude of the fluorescence changes during these various steps. Although our experiments do not allow us to determine all these parameters, especially because part of the fluorescence signal is lost within the dead-time of the stopped-flow equipment, they are maybe enough to make the following hypothesis.

Firstly, in the case of *5*,*6-Br*_*2*_*DDM*, the overall fluorescence quenching curves observed, with both a fast initial component and a subsequent slower component, can probably be accounted for by assuming initial rapid binding of *5*,*6-Br*_*2*_*DDM* to the external monolayer (within a few milliseconds, see Fig 5A), followed by a relatively slow flip-flop (over a few seconds at 85 μM free *5*,*6-Br*_*2*_*DDM*, see Fig 5C), as previously suggested for DDM [30]. The slower phase was less obvious in dissociation experiments than in binding experiments probably because of the relatively slow rate of flip-flop for *5*,*6-Br*_*2*_*DDM* in perturbed membranes which gets even slower when both membrane monolayers progressively become almost completely free of detergent. The flip-flop rate of DDM may indeed depends significantly on other membrane components around, including for example transmembrane proteins. It might also explain why biological membranes can be easily solubilized by DDM, while pure liposomes get solubilized much more slowly [13].

Secondly, in the case of *5*,*6-Br*_*2*_*C*_*12*_*E*_*8*_, because of the absence of such a slow phase in the fluorescence quenching or de-quenching traces, half times corresponding to all rate constants must be equal to or shorter than a few milliseconds, but our equipment and data themselves do not allow us to decide whether these few milliseconds correspond to the half time for flip-flop, or to the one for true dissociation or association. Our 1987 filtration experiments with multilayered liposomes excluded that C_12_E_8_ flip-flop could be considered as the rate-limiting step, but only when compared with the 300–400 ms time-scale considered in our initial work [8]. However, on the timescale of a few milliseconds only determined in the present experiments, flip-flop of C_12_E_8_ might well be the rate-limiting step: a half time of a few milliseconds for *5*,*6-Br*_*2*_*C*_*12*_*E*_*8*_ flip-flop, with even faster rates for “true” dissociation or binding, would provide a fair explanation for the apparent concentration-independence of observed rate constants in Fig 2. Theoretical computations of C_12_E_8_ binding and dissociation in model systems using molecular dynamics simulations on a millisecond time-scale might help to address this possibility, but at this stage the idea that flip-flop might be the rate-limiting step for the movements of both C_12_E_8_ and DDM (although at a much faster step in the case of C_12_E_8_), seems to be reasonable.

To summarize, use of brominated C_12_E_8_ combined with stopped-flow measurements, instead of rapid filtration experiments using radiolabelled C_12_E_8_, allowed us to reach a safer conclusion concerning the dissociation rate of C_12_E_8_ previously bound to SR membranes. In the case of C_12_E_8_, this dissociation is much faster (half time of a few milliseconds, or even shorter) than the one previously estimated from the earlier rapid filtration experiments [8]. In the case of DDM, the rate of binding to or dissociation from membrane is probably fast but a slower flip-flop rate significantly limits overall binding or dissociation. The present work therefore fully confirms the interest of using brominated detergents and the accompanying quenching of membrane protein intrinsic fluorescence for studying detergent/membrane interactions [9].

## Supporting information

S1 NC3Rs ARRIVE Guidelines Checklist (PDF)

## Abbreviations

*Br*_*2*_C_12_E_8_: octaethylene-glycol*-5*,*6-*dibromododecylether
C_12_E_8_: octaethylene-glycol*-*dodecylether
*Br*_*2*_DDM: 5,6-dibromo-β-dodecyl maltoside
DDM: β-dodecyl maltoside
SR: sarcoplasmic reticulum
SERCA1a: sarco-endoplasmic reticulum Ca^2+^-ATPase, isoform 1a
EGTA: Ethylene glycol-bis(2-aminoethylether)-*N*,*N*,*N′*,*N′*-tetraacetic acid.
